# Modulation of Hippocampal Circuits by Muscarinic and Nicotinic Receptors

**DOI:** 10.3389/fncir.2017.00102

**Published:** 2017-12-13

**Authors:** Holger Dannenberg, Kimberly Young, Michael Hasselmo

**Affiliations:** Center for Systems Neuroscience, Department of Psychological and Brain Sciences, Boston University, Boston, MA, United States

**Keywords:** acetylcholine, presynaptic inhibition, tonic depolarization, cholinergic fibers, volume transmission

## Abstract

This article provides a review of the effects of activation of muscarinic and nicotinic receptors on the physiological properties of circuits in the hippocampal formation. Previous articles have described detailed computational hypotheses about the role of cholinergic neuromodulation in enhancing the dynamics for encoding in cortical structures and the role of reduced cholinergic modulation in allowing consolidation of previously encoded information. This article will focus on addressing the broad scope of different modulatory effects observed within hippocampal circuits, highlighting the heterogeneity of cholinergic modulation in terms of the physiological effects of activation of muscarinic and nicotinic receptors and the heterogeneity of effects on different subclasses of neurons.

## Introduction

Besides its role as a neurotransmitter in the peripheral nervous system, acetylcholine (ACh) in the central nervous system mainly acts as a neuromodulator by modulation of neuronal excitability, presynaptic release probability, postsynaptic responsiveness and synaptic plasticity. Thereby, ACh plays an important role in modulating cortical circuit activity. Empirical evidence indicates a role for ACh in normal physiological cognitive functions including attention to sensory stimuli (Sarter et al., 2005; Pinto et al., 2013; Bloem et al., 2014), coding of location and movement speed, learning and memory (Haam and Yakel, 2017) as well as a substantial role in regulating transitions between waking and sleep states (Xu et al., 2015). Previous articles have described detailed computational hypotheses about the role of cholinergic neuromodulation in enhancing the dynamics for encoding in cortical structures (Hasselmo, 2006) and the role of reduced cholinergic modulation in allowing consolidation of previously encoded information (Hasselmo, 1999). Rather than focus on these computational hypotheses, we aim to emphasize the broad scope of experimental data concerning the heterogeneity of different circuits and the cellular level modulatory effects of muscarinic and nicotinic receptors that will need to be addressed in future computational hypotheses.

Overall, cortical ACh is believed to enhance the signal-to-noise ratio (Hasselmo et al., 1995; Yu and Dayan, 2005; Hasselmo, 2006; Minces et al., 2017) and thus cortical sensitivity to external stimuli including attention and cue detection (Gritton et al., 2016). It also changes network dynamics in the hippocampal formation to allow more efficient encoding of novel stimuli (Hasselmo, 2006) that might underlie the effects of ACh on learning and memory behavior observed in rodents and human studies.

Evidence for the involvement of ACh in behavioral states associated with arousal comes from microdialysis measurements of ACh concentrations in rat brain lysates. These experiments revealed increases of ACh concentrations in the cortex and hippocampal formation during active waking and high arousal associated with presentation of novel stimuli or fear stimuli in comparison to quiet waking (Marrosu et al., 1995; Acquas et al., 1996). Furthermore, hippocampal ACh levels increase when animals are exposed to a novel spatial environment (Aloisi et al., 1997; Ceccarelli et al., 1999; Giovannini et al., 2001; Bianchi et al., 2003).

Notably, these changes in ACh concentrations in the brain are associated with changes in the overall electrical activity of the brain. In the hippocampal formation, this change in network activity is most easily observable as changes in theta rhythmic oscillatory activity, i.e., local field potential oscillations in the frequency range between 6–12 Hz in rodents peaking around 7–8 Hz in the running animal. Theta oscillations have been also recorded in the medial temporal lobe of human patients before undergoing brain surgery for epilepsy treatment. These recordings show task-dependent increases in lower frequency theta power during navigation in virtual environments (Kahana et al., 1999; Ekstrom et al., 2005). Recent recordings in humans while in motion in a real world arena showed a correlation between theta oscillation frequency and movement speed (Bohbot et al., 2017), very similar to the situation observed in rodents.

We will therefore begin our discussion of cholinergic modulation of neurons in the hippocampus and associated cortical regions with a discussion of the regulation of theta oscillations by ACh and its implication for memory processes.

## Cholinergic Modulation of Theta Activity

Numerous experiments using different approaches including electrolytic lesions (Winson, 1978) or pharmacological inactivation (Chrobak et al., 1989) have shown that theta oscillations in the hippocampal formation are diminished when neuronal activity in the medial septum/diagonal band of Broca (MSDB) is inhibited. The MSDB consists of cholinergic, glutamatergic and GABAergic subpopulations with rhythmic activity of the Parvalbumin (PV^+^) GABAergic neurons leading hippocampal rhythmic activity (Hangya et al., 2009). Nevertheless, there is an association between theta oscillations and cholinergic activity. First, electrical stimulation of the medial septal area can induce theta oscillations in the hippocampal formation (Green and Arduini, 1954) and theta activity is correlated with cholinergic activity (Monmaur et al., 1997). Furthermore, cholinergic agonists can induce theta-rhythmic activity patterns in the *in vitro* hippocampal slice preparation (Konopacki et al., 1987). Second, inactivation of the MSDB cholinergic subpopulation by focal injections of atropine, a muscarinic acetylcholine receptor (mAChR) antagonist, into the MSDB inhibits a lower frequency component (4–6 Hz) of theta oscillations in rats and rabbits (Kramis et al., 1975). A second higher frequency component (8–12 Hz) of theta oscillations was not affected by focal atropine injections. These experiments revealed the existence of an atropine-sensitive or cholinergic activity-dependent theta component and an atropine-resistant higher frequency theta component, which was most prominent during movement and is believed to be primarily driven by entorhinal cortex (EC) activity. Third, more recent development of amperometric measurements of cholinergic activity, which work on the time-scale of seconds (Burmeister et al., 2008), demonstrate ACh release occurs over many seconds after the appearance of spontaneous or induced theta oscillations in urethane-anesthetized rats (Zhang et al., 2010). Fourth, optogenetic activation of cholinergic medial septal neurons induces hippocampal theta oscillations (Dannenberg et al., 2015) and suppresses sharp wave-ripple events (Vandecasteele et al., 2014), which are the hallmark of a hippocampal network state characterized by the absence of theta oscillations.

The vast majority of cholinergic fibers in the hippocampal formation arise from the innervation by the cholinergic MSDB projection neurons, which are also integrated in the MSDB network which paces hippocampal rhythmic activity. Thus, under physiological conditions, the increase of ACh concentrations in hippocampal (and cortical) structures coincides with changes in hippocampal rhythmic activity paced by the rhythmic activity of the GABAergic MSDB neurons (Stewart and Fox, 1990; Tóth et al., 1997; Varga et al., 2008; Hangya et al., 2009). In addition, *in vitro* data utilizing a complete septohippocampal preparation showed carbachol application-induced theta-like hippocampal oscillatory field potential activity that was synchronized with rhythmic IPSPs and rebound spiking in *I*_h_ expressing GABAergic MSDB neurons, which may generate or maintain theta rhythmic activity in the septohippocampal circuit (Manseau et al., 2008). We hypothesize that these changes act together to promote the processing and encoding of novel information into episodic memory. We will therefore briefly discuss the general function of theta oscillations for memory processing and then continue our discussion on the cellular effects of ACh and behavioral consequences.

Theta rhythmic activity is temporally structured and thus coordinates activity at different levels ranging from between-brain area synchronization to the organization of synaptic activity. The coordination of neuronal activity between brain regions has been shown for prefrontal cortex and hippocampus in spatial working memory tasks in rodents (Hyman et al., 2005; Jones and Wilson, 2005; Benchenane et al., 2010). Furthermore, between area phase synchronization of theta oscillations between visual cortex area V4 and the lateral prefrontal cortex have been observed in humans performing a visual memory task (Liebe et al., 2012). This synchronization on the local field potential level extended to the synchronization of spiking activity between V4 and the lateral prefrontal cortex. Similar spike-theta phase synchronizations have been shown between and within different brain regions in rodents (Skaggs et al., 1996; Lisman and Jensen, 2013) as well as in the medial temporal lobe in humans, where the tight coupling of spiking activity and the underlying theta oscillations predicted successful memory formation (Rutishauser et al., 2010). The temporal structure provided by theta oscillatory activity also provides temporal windows for local circuit computations (Mizuseki et al., 2009). The same theta frequency stimulus can induce both long-term potentiation (LTP) or long-term depression (LTD), depending on which phase of the theta oscillation it is delivered (Huerta and Lisman, 1995; Hyman et al., 2003). Taken together, these data show synaptic activity, spiking activity and local circuit computations differ at the peak and trough of local theta oscillations. These different activity patterns become even more obvious when looking at recordings of gamma activity (25–110 Hz) superimposed on theta oscillations. These data show that the magnitude and frequency of gamma oscillations change between the peak and trough phases of the theta cycle (Bragin et al., 1995; Belluscio et al., 2012). In hippocampal CA1, slow gamma (~25–55 Hz) occurs at the trough of local theta oscillations and is driven by input from the CA3 region, whereas fast gamma (~60–110 Hz) occurs around the peak of the local theta cycle and coincides with inputs from layer III medial EC (Colgin et al., 2009). The temporal coordination of entorhinal and CA3 inputs in concert with recruiting local inhibition controls spike timing in CA1 neurons and consequently phase precession in CA1 place cells, as shown recently by Fernández-Ruiz et al. (2017). Systemic administration of muscarinic receptor blockers weakens the phase relationship of gamma to theta in the EC (Newman et al., 2013). Finally, the different activity states temporally defined and separated by the opposite phases of the theta oscillation might reflect separate computational time windows for encoding and retrieval processes (Hasselmo et al., 2002) as discussed in greater detail below.

## Acetylcholine Effects on Memory

In humans, pharmacological disruption of cholinergic function by systemic administration of the muscarinic receptor antagonist scopolamine impairs new word encoding for subsequent free recall (Ghoneim and Mewaldt, 1977) or paired-associate learning (Atri et al., 2004). Systemic administration of scopolamine in humans impairs both object and spatial n-back working memory (Green et al., 2005). In the same study, simultaneous application of scopolamine and the nicotinic receptor antagonist mecamylamine produced even greater impairments, suggesting synergistic actions of muscarinic and nicotinic receptor activation for this kind of working memory. In rodents, pharmacological blockade of either hippocampal nicotinic receptors or M1 muscarinic receptors by local drug injections in rats impairs memory performance in 8-arm radial maze tasks (Ohno et al., 1993, 1994) and direct injection of scopolamine into the dorsal hippocampus impairs encoding of spatial information in the Morris water maze-task (Blokland et al., 1992). Importantly, local injections of scopolamine into the hippocampal CA3 or CA1 subfields in rats performing the modified Hebb-Williams maze-task selectively disrupted encoding of spatial information, while sparing retrieval (Rogers and Kesner, 2003). Conversely, enhancing ACh levels in CA3 or CA1 by local injections of the acetylcholinesterase (AChE) inhibitor physostigmine selectively disrupted retrieval, but spared encoding. In addition, activation of mAChR on apical dendrites of CA1 hippocampal pyramidal neurons leads to cytosolic calcium rises which acts to amplify nuclear calcium rises in response to trains of action potentials, modulating gene transcription (Power and Sah, 2002). Taken together with the positive effects of hippocampal ACh on synaptic plasticity and theta oscillations, these data favor a model in which high levels of ACh promote an encoding state of the entorhinal-hippocampal network.

## Cellular Effects of Acetylcholine

One possible mechanism contributing to the maintenance of information during working memory tasks as well as during the encoding of novel information is the intrinsic capacity of individual neurons to exhibit persistent spiking activity. This intrinsic persistent spiking has been demonstrated *in vitro* in neurons of the medial EC layer II (Klink and Alonso, 1997), medial EC superficial layer III (Yoshida et al., 2008) and deep layer V (Egorov et al., 2002), lateral EC layer III (Tahvildari et al., 2007), dorsal presubiculum (Yoshida and Hasselmo, 2009) as well as hippocampal subregions CA1 (Knauer et al., 2013) and CA3 (Jochems and Yoshida, 2013) in rats. *In vitro*, these neurons can fire for minutes after an initial depolarizing current injection, if the cholinergic agonist carbachol (Klink and Alonso, 1997; Egorov et al., 2002) or an agonist of the metabotropic glutamate receptor is present in the bath solution (Yoshida et al., 2008). Persistent spiking activity of neurons in layer V of the medial EC has been shown to be graded and can be maintained at different frequencies for many minutes (Egorov et al., 2002). This graded persistent firing could allow these neurons to integrate synaptic input over extended periods. Mechanistically, the induction of persistent spiking has been attributed to the activation of a Ca^2+^ sensitive cationic current (Jochems and Yoshida, 2013; Knauer et al., 2013).

These intrinsic cellular mechanisms could contribute to persistent spiking that has been observed *in vivo*, albeit the *in vivo* activity may depend on network dynamics. Recordings performed by Suzuki et al. (1997) show a sample-specific delay of activity in the EC during the delay intervals of a place memory task in macaques, and Young et al. (1997) observed prolonged odor-selective activity throughout or at the end of the memory delay period of an odor-guided delayed nonmatching-to-sample task in rats. Furthermore, recordings from head-direction cells in the dorsal presubiculum have shown that these neurons continue to spike when the animal’s head remains in the preferred direction of the cell (Taube and Muller, 1998), thus showing a very similar persistence of spiking activity. Graded persistent spiking activity is not limited to areas of the hippocampal formation, but can also be observed in other areas, such as the oculomotor system (Robinson, 1972), the somatosensory system (Romo et al., 1999), or the head direction system (Taube and Bassett, 2003). These cellular effects could contribute to network persistent activity observed during delayed matching tasks in human subjects as well (Schon et al., 2004). This persistent activity is reduced by systemic administration of muscarinic receptor blockers (Schon et al., 2005).

## Acetylcholine Receptors in the Hippocampal Formation

In area CA1 of the hippocampus, only approximately 7% of axon terminals from cholinergic neurons form synaptic junctional specializations (Umbriaco et al., 1995), and immunoelectron microscopic studies revealed a low frequency rate of synaptic membrane differentiations on choline acetyltransferase (ChAT)-immunostained cholinergic axon terminals in various regions of the CNS including the hippocampus, suggesting that diffuse transmission by ACh prevails in many regions of the CNS (Descarries et al., 1997). These anatomical data thus indicated that ACh is released in a manner described as volume transmission, i.e., ACh is released from the axonal terminals into the extracellular space. This view, however, was recently challenged by Takacs et al. (2017) demonstrating that all hippocampal cholinergic terminals establish synapses, and vesicles dock only at synapses. Nonetheless, ACh release in the hippocampal formation affects multiple cells and cellular compartments, which further contributes to network complexity. First, ACh acts via different subtypes, namely nicotinic and mAChR. Second, these receptors are differentially expressed on multiple interneuron subtypes and principal cells (Levey et al., 1995; Picciotto et al., 2012), as well as astrocytes (Van Der Zee et al., 1993; Sharma and Vijayaraghavan, 2001; Pabst et al., 2016). Third, these receptors are also found at different cellular compartments. It is therefore a difficult task to decipher the individual contributions of all these factors to the overall network effects of ACh. We will begin the discussion of the different cellular effects of ACh with a description of the nicotinic and muscarinic receptor types in the hippocampal formation and the functional consequences of their activation and will then summarize the cholinergic effects on the various interneuron subtypes in hippocampus and neocortical regions.

## Cellular Effects of Nicotinic Receptor Activation in Principal Neurons

The nicotinic acetylcholine receptor (nAChR) is an ionotropic receptor built as a homo- or heteromeric pentamer, which can be activated pharmacologically by the drug nicotine and functions as a non-selective, excitatory cation channel (Changeux et al., 1998; Picciotto et al., 2012). On a behavioral level, local infusion of nicotinic antagonists into the hippocampus results in spatial location memory impairment in rats (Ohno et al., 1993) highlighting the positive effect of nicotinic receptor activation on encoding. The predominant nAChR type in the hippocampus is the (α7)_5_ homomer (Séguéla et al., 1993; Radcliffe et al., 1999), followed in expression levels by the heteromeric (α4)_2_(β2)_3_ and (α3)_2_(β4)_3_ channel compositions (Zoli et al., 1998; Radcliffe et al., 1999).

Data from early experiments using intracellular recordings in guinea-pig hippocampal slices (Benardo and Prince, 1982a, b) demonstrated that perfusion of slices with medium containing the muscarinic antagonists atropine or scopolamine blocked the majority of ACh actions on CA1 pyramidal cell membrane potential changes pointing to a dominant role of muscarinic, but not nicotinic, receptors in modulation of cellular excitability. However, functional calcium imaging with Fura-2-acetoxymethyl ester revealed functional α7 nAChRs in CA3 principal cells as well as dentate gyrus (DG) granule cells (Grybko et al., 2010). Likewise, in region CA1 of rat hippocampal slices *in vitro*, stimulation with choline, which is a selective α7 nAChR agonist, in combination with an allosteric modulator of α7 nAChRs evoked small but reliable membrane depolarizations of about 4 mV (Kalappa et al., 2010). Taken together, these experiments provide evidence for functional somato-dendritic α7 nAChRs on DG granule as well as hippocampal pyramidal cells.

Importantly, there is a strong functional expression of α7 nAChRs on glutamatergic presynaptic terminals inside region CA3 (Gray et al., 1996), which can enhance the release of glutamate via protein kinase A activation (Cheng and Yakel, 2014). Activation of these receptors induced high-frequency bursts of miniature excitatory postsynaptic currents (mEPSCs) in CA3 pyramidal cells in rat hippocampal slices (Gray et al., 1996; Sharma and Vijayaraghavan, 2003). Such mEPSCs were sufficient to drive postsynaptic spiking in the absence of incoming action potentials, which were inhibited by tetrodotoxin application (Sharma and Vijayaraghavan, 2003). Consistent with these observations, nicotine has been demonstrated *in vitro* to increase intracellular Ca^2+^ in mossy fiber presynaptic terminals and to enhance the frequency of mEPSCs, as well as miniature inhibitory postsynaptic currents (mIPSCs) recorded from CA3 neurons in rat hippocampal slices (Radcliffe et al., 1999). Likewise, nicotine application caused a short initial reduction followed by a longer period of enhancement of stimulation-induced field excitatory postsynaptic potential (EPSP) amplitudes (Giocomo and Hasselmo, 2005). This effect was selective for stratum lacunosum-moleculare, and was absent in stratum radiatum of CA3. However, this effect was blocked by GABA antagonists indicating it was mediated by effects on GABAergic transmission. These results indicate selective nicotinic receptor-mediated enhancement of afferent inputs to hippocampal CA3, whereas recurrent excitation appears to remain unaffected. The nicotinic enhancement of synaptic transmission in the hippocampal formation is consistent with the proposed role of nicotinic receptors in enhancing thalamic input to neocortical structures (Gil et al., 1997; Disney et al., 2007) and the output of cortical neurons (Poorthuis et al., 2013). These network effects of nicotine in neocortical structures may serve to enhance mechanisms of attention (McGaughy and Sarter, 1998; Bloem et al., 2014).

## Cellular Effects of Muscarinic Acetylcholine Receptor Activation in Principal Neurons

In contrast to the ionotropic nature of the nicotinic AChR type, the muscarinic AChR, which is activated by the drug muscarine, is metabotropic, i.e., it acts via functional coupling and activation of heteromeric G proteins. Five subtypes of mAChR have been identified and termed M1–5. The receptor types M1, M3 and M5 are coupled to G_q_ proteins which activate phospholipase C, which leads to Ca^2+^ influx and activation of intracellular signaling cascades. In contrast, M2 and M4 receptors are coupled to G_i/o_ proteins, that inhibit the enzyme adenylyl cyclase and thereby reduce the production of cAMP (Wess, 2003). A quantification of relative proportions of the M1–M5 mAChR subtypes with immunoprecipitation followed by a radioligand binding assay in post-mortem tissue of the human hippocampus found about 60% M1, 20% M2, 15% M4 and roughly 5% M3 receptor expression (Flynn et al., 1995). Similarly, the same method applied to rat hippocampal tissue revealed a proportion of about 36% M1, 33% M2 and 27% M4 receptor expression. M3 receptors were not examined (Levey et al., 1995). Although M5 mRNA can be detected by *in situ* hybridization in CA1 pyramidal cells of the rat hippocampus (Vilaró et al., 1990), the protein expression is very low (Wall et al., 1992) with unknown functional significance. Hence, M1, M2, and M4 are the most prevalent receptor subtypes in the hippocampus. The expression of the M2 muscarinic subtype is restricted to interneurons, thus is not present on principal cells (Levey et al., 1995). M1 is widely distributed within the hippocampus and preferentially expressed in somata and dendrites of hippocampal pyramidal and DG granule cells (Levey et al., 1995; Yamasaki et al., 2010), with only a small fraction expressed on axons and terminals. From a functional perspective, M1 receptors are mainly responsible for the modulation of pyramidal cell excitability upon transient/phasic ACh application in slices (Gulledge and Kawaguchi, 2007).

Pioneering studies of cholinergic effects on hippocampal pyramidal cells using intracellular recordings in combination with pharmacology utilizing the *in vitro* guinea pig hippocampal slice preparation by Benardo and Prince (1982a) demonstrated a slow and long-lasting depolarization of CA1 pyramidal neurons and increases in spike frequency upon drop or iontophoretic application of ACh to the dendritic region in stratum oriens and stratum radiatum. The observed muscarinic cholinergic depolarization occurred as a result of blockade of voltage-dependent K conductance (distinct from that of the delayed rectifier; Benardo and Prince, 1982c) resembling the M-current initially discovered in bullfrog sympathetic ganglion cells (Brown and Adams, 1980). Additionally, these findings are consistent with subsequent studies utilizing rat hippocampal slices showing a slow depolarization of pyramidal cells via cholinergic modulation through suppression of a leak potassium current (Cole and Nicoll, 1984).

Interestingly, CA1 and CA3 principal cells respond differently to such phasic local ACh puff applications. ACh application near the somata of CA1 principal cells resulted in a 2.5 mV hyperpolarization of the membrane potential and inhibited action potential generation via calcium dependent activation of small conductance calcium activated potassium (SK) channels, Dasari and Gulledge (2011) while ACh applied focally to CA3 principal cell somata generated a small depolarization of about 0.6 mV. In contrast to phasic application, tonic cholinergic modulation of CA1 principal cells via application of carbachol decreases medium afterhyperpolarizations (AHPs) and the early component of the slow AHPs, generated afterdepolarizations (ADPs) via M1 mAChRs, and depolarized CA1 principal neurons via M1 and M3 mAChRs (Dasari and Gulledge, 2011) consistent with previous studies (Madison and Nicoll, 1984; Madison et al., 1987) showing a reduction of spike frequency accommodation due to a blockade of calcium-activated potassium slow AHPs.

Besides M1, M4 is the other major mAChR subtype responsible for direct cholinergic modulation of the excitatory hippocampal circuit. In contrast to the preferential somato-dendritic localization of M1, M4 is mainly located in glutamatergic terminals and mediates cholinergic presynaptic inhibition of Schaffer collateral EPSPs *in vitro* (Shirey et al., 2008; Dasari and Gulledge, 2011). As reviewed previously (Hasselmo, 2006), the cholinergic presynaptic inhibition of glutamatergic synaptic transmission in the hippocampus has been shown in a wide range of studies *in vitro* (Hounsgaard, 1978; Valentino and Dingledine, 1981; Lambert and Teyler, 1991) and *in vivo* utilizing recordings of CA1 field potential responses evoked by ipsilateral CA3 (Herreras et al., 1988) or commissural stimulation in anesthetized rats (Rovira et al., 1983), consistent with earlier findings by Leung and Vanderwolf (1980) showing that injections of atropine sulfate severely dampen the oscillations in averaged evoked potentials in CA1 of rats during walking or similar movements. ACh mediates a stronger presynaptic inhibition of the midapical than basal and distal apical excitation (Leung and Péloquin, 2010) and presynaptic muscarinic inhibition has been shown to be stronger at excitatory recurrent connections and the Schaffer collaterals compared to the afferent input from EC (Hasselmo and Schnell, 1994; Hasselmo et al., 1995).

## Cellular Effects of Nicotinic Receptor Activation in Interneurons

In contrast to pyramidal and granule cells, interneurons can have very large nAChR currents with fast kinetics. However, these effects vary substantially between different neuronal subtypes within the hippocampal formation (McQuiston and Madison, 1999c; McQuiston, 2014). Activation of nicotinic currents, mainly mediated by the α7 subtype, have been observed in recordings of stratum radiatum interneurons in the hippocampus (Jones and Yakel, 1997; Frazier et al., 1998; McQuiston and Madison, 1999c) and in DG molecular layer interneurons, hilar interneurons and the glutamatergic mossy cells of the dentate hilus (Frazier et al., 2003). These currents have been also observed in oriens-lacunosum moleculare (O-LM) interneurons in rat hippocampal slices (Alkondon et al., 1999; McQuiston and Madison, 1999c). In addition, Alkondon et al. (1999) also found slow but long-lasting depolarizations mediated by activation of α4β2 nicotinic receptors in both stratum radiatum and O-LM interneurons. In contrast to these experiments, which used pressure injections of ACh, a more recent study from Bell et al. (2011) used optogenetics to trigger synaptic release of ACh. Similar to the results by Alkondon et al. (1999), they found that optogenetic activation of cholinergic fibers resulted in mostly subthreshold depolarizations with slow kinetics mediated by the activation of α4β2 nicotinic receptors in CA1 O-LM interneurons. However, they did not find α7 nAChR mediated currents in O-LM interneurons, raising the possibility that the type of stimulation (optogenetic vs. pressure injection) might bias activation of nicotinic receptor subtypes. Nevertheless, experimental data indicates that, in general, any of the individual interneuron morphological subtypes can be modulated by all three nAChR subtypes (Alkondon and Albuquerque, 2004). A significant portion of the network effects of ACh likely results from the modulation of specific subtypes of inhibitory interneurons, which are in a powerful position to control rhythmic activity, and synaptic inputs to and spiking output from pyramidal neurons (McQuiston and Madison, 1999a; McQuiston, 2014).

## Cellular Effects of Muscarinic Acetylcholine Receptor Activation in Interneurons

In contrast to the mainly slow depolarizing synaptic response mediated by mAChR activation in principal cells, hippocampal interneurons respond with a much greater diversity regarding the waveform of synaptic potentials, as shown for CA1 interneurons in rat hippocampal slices with electrical (McQuiston and Madison, 1999a; Widmer et al., 2006) or optogenetic stimulation of synaptic ACh release (Bell et al., 2013). Benardo and Prince (1982a) provided one of the earliest observations of ACh-mediated excitation of hippocampal inhibitory interneurons and subsequent inhibition of pyramidal neurons in guinea pig slices. Cholinergic excitation of hippocampal interneurons was later confirmed using recordings in rat hippocampal slices, and this excitation was mostly blocked by muscarinic antagonists (Reece and Schwartzkroin, 1991).

Consistently, interneurons that responded to optogenetically released ACh predominantly had muscarinic (80%) vs. nicotinic (17%) mediated changes in membrane potential, and only 3% of interneurons had mixed responses (Bell et al., 2013). In the studies from Widmer et al. (2006) and Bell et al. (2013) the majority of interneurons (64% and 40%, respectively) responded with an atropine-sensitive (i.e., muscarinic) slow depolarization upon synaptic ACh release. In those studies, 13% and 25%, respectively, responded with a biphasic hyperpolarization and depolarization, and 20% and 35%, respectively, showed a pure hyperpolarizing response. Depolarization of hippocampal interneurons by bath application of carbachol, a cholinergic agonist, can induce theta-frequency membrane potential oscillations *in vitro*, which has been suggested to contribute to intrinsically generated theta-rhythmic firing of pyramidal neurons due to rebound spiking (Chapman and Lacaille, 1999). However, only a minor fraction of cells in the study by Widmer et al. (2006) responded with membrane potential oscillations (2%) without any obvious correlation to a morphological classification of the different interneurons. In the study by Bell et al. (2013), the hyperpolarizing response observed in a subset of interneurons was demonstrated to be mediated via activation of an inwardly rectifying potassium channel by activation of the M4 mAChR subtype, whereas the depolarizations were likely produced by M3 receptor activation. Considering the correlation of ACh levels with different functional network states, it is noteworthy that hyperpolarizing responses required less optogenetic stimulation strength, i.e., less synaptic ACh release, than depolarizing responses. This favors a model proposed by McQuiston (2014), in which low levels of ACh favor disinhibition, whereas higher levels of ACh favor inhibition of hippocampal principal cells. If interneurons are depolarized by M1/M3 mAChR activation, this can lead to consistently enhanced firing frequency and the production of ADPs, as shown for O-LM interneurons (Lawrence et al., 2006b) as well as basket cells (Cea-del Rio et al., 2010) in CA1. Concomitantly, mAChR activation enhances firing reliability and precision to theta frequency input in O-LM (Lawrence et al., 2006a) as well as Cholecystokinin (CCK^+^) positive Schaffer collateral associated and basket cells (Cea-del Rio et al., 2011). Despite the effect of increasing IPSC frequency in CA1 and CA3 pyramidal neurons, activation by carbachol significantly decreased the amplitude of monosynaptically evoked IPSCs mediated by perisomatic inhibitory interneurons indicating postsynaptic depolarization of interneurons is combined with the presynaptic inhibition of inhibitory transmitter release (Pitler and Alger, 1992; Behrends and ten Bruggencate, 1993; Szabó et al., 2010). Presynaptic M2-type mAChRs were responsible for the reduction in IPSC amplitude in CA3 axo-axonic and PV^+^ basket cell-pyramidal cell pairs, whereas postsynaptic M1/M3 receptors in pyramidal cells triggered the synthesis of endocannabinoids, which activated type 1 cannabinoid receptors (CB1) at the terminals of CCK^+^ basket cells, resulting in reduced GABA release (Fukudome et al., 2004; Szabó et al., 2010). Besides their abundant presynaptic location on inhibitory terminals, M2 mAChRs are also expressed postsynaptically in dendrites and somata of hippocampal interneurons located inside or close to stratum oriens as well as hilar interneurons (Hájos et al., 1998; Rouse et al., 1998). Furthermore, M2 mAChRs are located not only on non-cholinergic, but also cholinergic terminals (Rouse et al., 2000), where they can function as presynaptic autoreceptors, the activation of which inhibits ACh release, as shown in synaptosomes from rat hippocampus (Raiteri et al., 1984).

## Cholinergic Effects Based on Molecular Markers for Interneuron Subtypes

Interneurons display an immense functional, morphological and genetic diversity, as described in previous reviews of the classification of interneuron subtypes (McBain and Fisahn, 2001; Somogyi and Klausberger, 2005; Rudy et al., 2011; Kepecs and Fishell, 2014). The previous sections focused on different morphological subtypes identified by anatomical features of the neurons, whereas this section will focus on differences in cholinergic effects based on different molecular markers for interneurons. Distinct expression profiles of neuropeptides that relate to points of synaptic contact, morphological structure and intrinsic excitability (Freund and Buzsáki, 1996; Kepecs and Fishell, 2014) distinguish interneurons. These characteristics contribute to shaping the spike timing of downstream neurons (Klausberger and Somogyi, 2008), mediating synchronization and rhythmicity (Cobb et al., 1995) with respect to local ongoing network rhythms, and balancing the excitatory gain through divisive (or arrhythmic) inhibitory control. Their pivotal role in local computation lies in their ability to innervate specific sublaminar populations as well as subcellular compartments. Thus, cholinergic modulation of interneuron activity can have strong influences on local network computations. However, there is still a limited understanding of the functional role of the differential cholinergic responses in the different subtypes of interneurons. Here, we summarize some of the current observations of cholinergic effects on different interneuron subtypes in hippocampus and neocortex.

Interneurons expressing the calcium binding protein parvalbumin (PV) make up approximately 40% of all GABAergic interneurons. However, this is a heterogeneous group of functionally distinct interneuron subtypes. For example, in the hippocampus alone, there are at least three functionally and morphologically distinct populations of PV^+^ expressing interneurons, namely basket, axo-axonic and bistratified cells. Fast spiking interneurons in the hippocampus and neocortex are often PV^+^ positive and target the soma of pyramidal cells. In general, these fast spiking PV^+^ interneurons elicit a range of responses to muscarinic activation in different brain areas. In the frontal cortex fast firing PV^+^ cells have been shown to be unresponsive to mAchR activation (Kawaguchi, 1997; Gulledge et al., 2007). In the visual cortex, however, PV^+^ cells have been demonstrated to show mildly hyperpolarizing changes in membrane potential in response to mAchR activation (Xiang et al., 1998). Muscarinic receptors cause presynaptic inhibition of GABA release from fast firing interneurons in thalamocortical slices that contrasts with nicotinic-mediated enhancement of thalamocortical excitatory inputs on pyramidal neurons (Kruglikov and Rudy, 2008). In contrast, a mixture of depolarizing, hyperpolarizing and biphasic responses to muscarinic activation of PV^+^ basket cells have been observed in slices of the hippocampus (Bell et al., 2013; Yi et al., 2014). In addition to changes in baseline resting membrane potential, hippocampal PV^+^ basket cells have also been shown to increase their firing frequency during cholinergic modulation. Trains of action potentials elicit slow afterhyperpolarization potentials (sAHPs) in PV^+^ basket cells, and these sAHPs are reduced by bath application of muscarine (Cea-del Rio et al., 2010). Furthermore, the AHPs following action potentials and the membrane depolarization following cholinergic activation observed in hippocampal PV^+^ basket cells have been demonstrated to rely on M1 muscarinic receptor expression (Cea-del Rio et al., 2010), and M1 gene knockout produces complex but selective memory deficits in mice (Yi et al., 2014). Taken together, despite many reports of unresponsive or mild effects of ACh on PV^+^ cells, evidence points to hippocampal and prefrontal PV^+^ interneurons exhibiting direct depolarization from muscarinic activation (McBain et al., 1994; Chiang et al., 2010).

Muscarinic activation of PV^+^ interneurons has been observed to increase the frequency of IPSPs in downstream pyramidal cells, but these effects vary greatly depending on brain region and cortical layer. Postsynaptic observations of IPSPs in hippocampus show that muscarinic modulation increases the frequency of spontaneous IPSPs but decreases the amplitude of evoked IPSCs (Pitler and Alger, 1992; Behrends and ten Bruggencate, 1993). This indicates postsynaptic depolarization of interneurons is combined with presynaptic inhibition of the release of GABA. The recruitment of PV^+^ cells in hippocampus and prefrontal cortex during brain states exhibiting high levels of ACh has been linked to the recognition of novel objects and spatial aspects of working memory (Yi et al., 2014). In the visual cortex, ACh levels have been associated with the regulation of PV^+^ cells for altering gain control during attentive behavioral states such as locomotion (Fu et al., 2014), although this regulation is likely indirect through a disinhibitory circuit involving vasoactive intestinal peptide (VIP) positive cells.

Another neurochemically defined group of interneurons are somatostatin (SST) positive interneurons (Rudy et al., 2011). This group represents about 30% of GABAergic neurons in the brain including Martinotti cells in the neocortex, and O-LM and bistratified cells in the hippocampus. Martinotti cells in the neocortex and O-LM cells in the hippocampus selectively innervate the dendrites, rather than the perisomatic region of downstream principal cells, but also synapse onto other non-SST^+^ inhibitory interneurons. Muscarinic agonists have been shown to produce transitions from AHPs to ADPs in O-LM cells which can result in persistent spiking (McQuiston and Madison, 1999b; Lawrence et al., 2006b). Functionally, these ADPs have been linked to the increase in firing response of stratum oriens interneurons to theta frequency inputs (Lawrence et al., 2006a). O-LM cells have been implicated in controlling the inputs to CA1 pyramidal cells, differentially suppressing extrahippocampal (entorhinal cortical) inputs at the distal apical dendrites of pyramidal cells, whilst facilitating inputs from CA3 to proximal dendrites (Leão et al., 2012).

The serotonin receptor 5HT3a expressing group of interneurons, which includes the population of VIP^+^ expressing interneurons as well as the separate population of neurogliaform cells, make up for the remaining 30% of GABAergic interneurons (Rudy et al., 2011). VIP^+^ interneurons express both nACHRs and ionotropic serotonergic receptors, suggesting that these neurons mediate rapid changes due to input from neuromodulators. VIP^+^ interneurons form a particularly interesting population, because they mainly target other interneurons including the PV^+^ and SST^+^ interneurons (Pfeffer et al., 2013) and express both nAChRs as well as ionotropic serotonergic receptors suggesting that these neurons are key for mediating rapid changes from neuromodulators. Indeed, the class of VIP^+^ interneurons has been suggested to mediate disinhibitory control in multiple areas of the neocortex (Pi et al., 2013; Fu et al., 2014), and this effect has been proposed to be due to fast nicotinic activation of VIP^+^ neurons (Fu et al., 2014). Muscarinic receptors also act to depolarize VIP^+^ interneurons (Bell et al., 2015). The cholinergic depolarization of VIP^+^ interneurons has been proposed to cause indirect disinhibition of principal cells via an increase in inhibition onto downstream PV^+^ and SST^+^ cells. *In vivo* experiments show that VIP^+^ interneuron activity correlates with behavioral recognition of sensory cues (Kuchibhotla et al., 2017) and probably acts to convey information about reinforcement events and behavioral context. Interestingly, in these experiments, optogenetic activation of a very small minority of VIP^+^ cells (1%–2%) led to the recruitment of nearly 20% of excitatory cells demonstrating the powerful extent of VIP^+^ activity on the local network. There is still some debate as to whether this disinhibition acts more globally or more locally, since there is more recent evidence that VIP^+^ cells project within a narrow vertical column and serve to “open holes in the blanket of inhibition” (Karnani et al., 2016).

CCK^+^ interneurons cells are also within the grouping of 5HT3a expressing inhibitory interneurons, and form a second functional class of perisomatically targeting basket cells. Interestingly, PV^+^ basket cells express M1 mAChR mRNA, but entirely lack M3 mRNA, whereas CCK^+^ basket cells show robust expression of both M1 and M3 mRNA. The additional expression of M3 makes the CCK^+^ basket cells more sensitive than PV^+^ basket cells for increases in firing rates upon cholinergic input, as shown in CA1 of mouse hippocampal slices (Cea-del Rio et al., 2010). In line with the activation of perisomatic inhibitory interneurons, optogenetically released ACh resulted in an increase of IPSCs onto CA1 pyramidal neurons (Nagode et al., 2011; Bell et al., 2015). Interestingly, the theta-rhythmic IPSCs could be blocked by endocannabinoid release from pyramidal cells, providing further support that the main source of IPSCs are CB1 expressing CCK^+^ basket cells (Nagode et al., 2011, 2014). Dendritically projecting Schaffer collateral-associated CCK^+^ cells, which shape dendritic excitability and synaptic integration, showed similar changes in excitability, except that they showed a biphasic change corresponding to an initial M1-mediated hyperpolarization, followed by an M3-mediated depolarization of their membrane potential (Cea-del Rio et al., 2011).

An important line of research has shown that CB1 expressing CCK^+^ basket cells strongly demonstrate the phenomenon of depolarization induced suppression of inhibition (DSI), a retrograde signaling mechanism, where endocannabinoids are released from depolarized pyramidal cells act on upstream CB1 receptors on CCK^+^ basket cells to transiently reduce the frequency of GABA vesicle release. Notably, this phenomenon is highly accentuated when muscarinic receptors on CCK^+^ basket cells cause larger than baseline IPSP frequencies and postsynaptic depolarization amplitudes in CA1 pyramidal cells (Martin and Alger, 1999). Interestingly, activation of M1/M3 receptors also causes an increase in endocannabinoid production (Fukudome et al., 2004). The synergistic interaction between the endocannabinoid and cholinergic systems at presynaptic CCK^+^ terminals may be modulating the timing and frequency of GABA release onto CA1 pyramids. In turn, the frequency of phasic inhibition may be important for temporal entrainment of CA1 pyramidal cells with respect to ongoing network rhythms, and more generally coordinating downstream pyramidal spike timing (Pouille and Scanziani, 2001; Daw et al., 2009; Alger et al., 2014).

In summary, the subtype-specific cholinergic modulation of interneuron activity can have strong influences on network dynamics in the hippocampus and other cortical structures. Evidence for this was given recently by an *in vivo* study from Lovett-Barron et al. (2014), in which aversive stimuli were shown to activate CA1 O-LM interneurons via cholinergic input, leading to inhibition of the distal dendrites of CA1 principal cells, which was necessary for successful fear learning.

## Cholinergic Control of Network Dynamics

**Figure 1:**
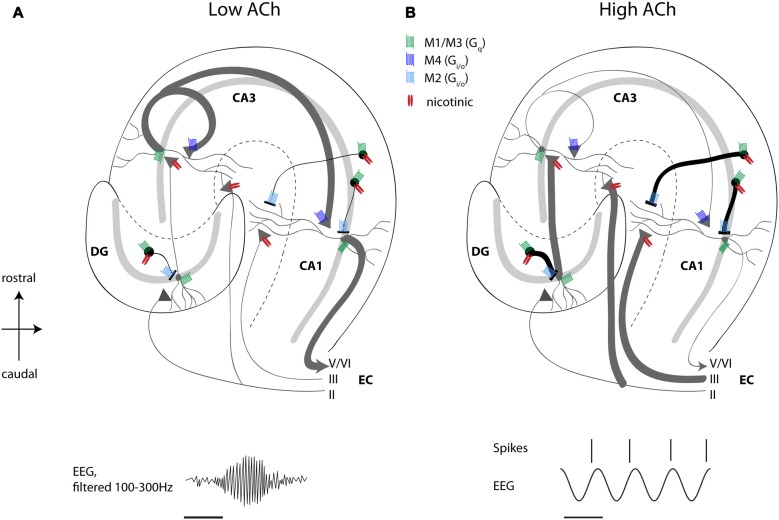
High acetylcholine (ACh) levels enhance encoding and suppress consolidation dynamics in the hippocampus. Schematic drawing of a transverse slice of hippocampus with the main circuit connections and locations of muscarinic and nicotinic receptors shown. **(A)** When ACh release is low, recurrent excitatory hippocampal activity leads to retrieval and consolidation of previously stored information, which can support the consolidation of memory during sharp wave ripples activity (ripple event schematically depicted in lower left panel). **(B)** High ACh levels result in nicotinic enhancement of mossy fiber and perforant path inputs, thereby potentiating afferent input synapses in the hippocampus, which favors the encoding of novel information. At the same time, muscarinic depolarization of interneurons and muscarinic presynaptic inhibition of synaptic potentials at recurrent and Schaffer collateral synapses result in suppression of recurrent excitation associated with retrieval of information. Concomitantly, muscarinic and nicotinic excitation of interneurons results in reduced, but temporally more precise spiking activity of pyramidal cells during ongoing theta oscillations (schematically depicted in lower right panel). See text for a more detailed description of receptor distributions and functions. Scale bar for EEG, 125 ms. Principal cells with dendrites schematically depicted in gray, black circles represent interneurons, triangles represent synaptic terminals. ACh, acetylcholine; DG, dentate gyrus; CA, cornu ammonis; EC, entorhinal cortex; EEG, electroencephalogram.

One striking cellular effect of increasing cholinergic activity is the enhancement of the influence of feedforward afferent input while simultaneously suppressing the influence of excitatory feedback connections (Hasselmo, 2006). This has been discussed extensively in previous reviews (Hasselmo, 2006) but is briefly reviewed here. Physiological studies have shown an enhancement of afferent input caused by nicotinic receptor activation, and the presynaptic inhibition of excitatory feedback connections has been shown to be caused by M4 muscarinic presynaptic inhibition (Dasari and Gulledge, 2011). The selective presynaptic inhibition of recurrent excitation might provide a solution to the problem of proactive interference, which occurs when novel information has to be encoded in the same network capable of retrieving previously stored memories (Hasselmo, 2006). For hippocampal CA1, muscarinic presynaptic inhibition suppresses excitatory projections from CA3 to CA1, but spares inputs from medial EC, allowing a dominant influence of afferent input. Consistent with this model of muscarinic presynaptic inhibition in the hippocampus, local infusion of cholinergic antagonists in hippocampus causes an increase in background spiking activity in unit recordings (Brazhnik et al., 2003). Thereby, ACh regulates the spread of excitatory activity within hippocampal and cortical circuits. These findings match with data from *in vitro* studies showing inhibition of CA1 pyramidal neurons by ACh-mediated excitation of interneurons (Benardo and Prince, 1982a) and inhibition of DG granule cells due to muscarinic amplification of fast excitation in hilar neurons (Brunner and Misgeld, 1994), and more recent data from single unit recordings in the hippocampus and DG showing decreased spiking activity of pyramidal neurons and DG granule cell coinciding with a higher temporal precision of that spiking activity during optogenetic activation of cholinergic MSDB neurons (Dannenberg et al., 2015; Pabst et al., 2016). As outlined above, microdialysis studies show that cholinergic activity is low during quiet waking or slow wave sleep. This would release the presynaptic inhibition of excitatory feedback, allowing increased generation of sharp wave ripple activity (Hasselmo, 1999; Vandecasteele et al., 2014). The activity of medial septal neurons is indeed decreased during sharp wave ripple events (Dragoi et al., 1999) allowing a strong influence of consolidation based on previously modified recurrent connections. Overall, these data support the view that a primary function of septo-hippocampal ACh is to reduce interference in the learning process by adaptively timing and separating encoding from retrieval and consolidation processes (Figure 1).

In addition to the modulation by cholinergic presynaptic inhibition of synaptic transmission, the dynamics of theta rhythm might also contribute to the separation of encoding and retrieval (Hasselmo et al., 2002). The trough of the local theta oscillation in stratum pyramidale is associated with hyperpolarization of the soma of hippocampal pyramidal cells (Kamondi et al., 1998), which could prevent postsynaptic spiking that mediates retrieval of memories previously stored in the autoassociative CA3 network (Hasselmo et al., 2002), while allowing novel sensory input from the EC to depolarize dendrites at the same time, inducing synaptic modification. In the light of the experimentally found theta phase-dependent synaptic plasticity (see above), this restricts LTP to the synaptic contacts active during entorhinal inputs at the theta peak. This model of separate phase of encoding and retrieval is supported by experimental data showing that CA1 ensemble firing in rats shifts closer to the theta peak in a novel environment, but scopolamine injections shift the ensemble firing closer to the trough (Douchamps et al., 2013). Further supporting experimental data comes from a study by Siegle and Wilson (2014) who showed that inhibition of CA1 principal cells by optogenetic stimulation of PV^+^ interneurons could either support encoding or retrieval in an end-to-end T maze task when inhibition was targeted to the putative retrieval or encoding cycle of the theta oscillation, respectively (Siegle and Wilson, 2014). Lower levels of ACh release the presynaptic inhibition of excitatory feedback within cortical structures (Hasselmo, 1999), which allows a stronger influence of hippocampus on neocortex that could underlie the consolidation of previously encoded memories (Dewar et al., 2014; Craig et al., 2016).

## Cholinergic Modulation of Location Coding

Numerous studies have shown robust impairments of spatial memory behavior after lesions of the medial septum (Winson, 1978; Givens and Olton, 1990). Similarly, recordings of place cells in rats after lesions of the fimbria-fornix, the main fiber track containing septo-hippocampal projection fibers from cholinergic and other medial septal neurons, have revealed a reduction in the spatial specificity and reliability of firing of place cells, and have shown that place cells are more sensitive to maze rotation (Shapiro et al., 1989). However, lesions of the MSDB or the fimbria-fornix are not specific to the cholinergic subpopulation of medial septum neurons. But the development of an immunotoxin composed of the neurotoxin saporin coupled to an antibody against the nerve growth factor receptor, which is enriched on cholinergic neurons in the MSDB, allowed more specific lesions of the cholinergic subpopulation within the MSDB (Book et al., 1994). The behavioral effects of these selective lesions of cholinergic innervation appeared to be weaker than the effect of medial septum lesions (Parent and Baxter, 2004), but, interestingly, specifically influenced memory for the spatial location of objects in a where-which task in rats (Easton et al., 2011). In mice, saporin lesions of cholinergic MSDB neurons have been shown to cause impairments in the recognition of the spatial location of objects (Cai et al., 2012). Taken together, these data support the view that cholinergic signaling is important for the rapid updating of place cells when visual cues or object locations differ across spatial contexts or when spatial locations are relevant to memory-guided behavior.

## Cholinergic Signaling Associated with Spatial Novelty

ACh levels have been shown to increase during learning of a spatial memory task (Stancampiano et al., 1999) and during object exploration (Stanley et al., 2012). Furthermore, focal injection of scopolamine, a muscarinic receptor antagonist, degrades the place fields of hippocampal place cells, which is mostly reversible (Brazhnik et al., 2003). Exposing animals to a novel spatial environment not only increases hippocampal ACh concentrations, but also reduces the frequency of hippocampal theta oscillations, an effect slowly disappearing with increasing familiarity (Jeewajee et al., 2008) providing further hints that ACh modulates hippocampal network activity to better match the environmental demand for processing novel behaviorally relevant information. As outlined above, theta oscillations consist of a slower frequency atropine-sensitive component and a higher frequency atropine-resistant component, also known as type II and type I theta. Thus, the shift to lower frequency theta oscillations during novelty exposure can be explained by higher cholinergic activity, which overall helps integrating sensory experiences into episodic memory. Cholinergic modulation could also be relevant to the effect of novelty on the firing properties of grid cells and place cells. Recordings from rats exploring a novel environment show a larger spacing between the firing fields exhibited by each individual grid cell compared to the baseline spacing observed in a familiar environment (Barry et al., 2012a,b). This expansion of spacing could underlie the shifts in the firing location of place cells (termed remapping) that occurs in novel environments. This expansion of spacing has been proposed to arise from the increase of ACh levels in novel environments (Barry et al., 2012b).

## Effects of Acetylcholine on Synaptic Plasticity

Because ACh affects memory and learning, the question arises, how ACh modulates synaptic plasticity, which is generally assumed to be the cellular and molecular correlate of learning. A seminal article by Williams and Johnston (1988) showed muscarinic depression of LTP at the mossy fiber-CA3 synapse. Moreover, the same stimulus can induce either LTP or LTD depending on the precise timing in relation to the ongoing theta oscillation. One study analyzed these effects in slice preparations in which theta rhythm oscillations were induced by cholinergic modulation (Huerta and Lisman, 1995). In this study, a single burst given *in vitro* at the peak of theta measured in stratum radiatum near to the pyramidal cell layer induced homosynaptic LTP, whereas the same stimulus given at the theta trough induced homosynaptic LTD (Huerta and Lisman, 1995). These results were later confirmed with an experiment using similar burst stimulation in awake behaving rats (Hyman et al., 2003). In addition, single burst stimulation-induced LTP at basal dendrites of CA1 was significantly larger when it was induced during walking than during awake immobility, slow wave sleep, or REM sleep of rats (Leung et al., 2003). On the receptor level, pre- and postsynaptic nAChR and mAChR activity on principal cells and interneurons are involved in the modulation of synaptic plasticity in a complex manner. For instance, nAChR activity could enhance or depress synaptic plasticity with the form of the modulation depending on the location and timing of the nAChR activity relative to the electrical stimulation used for LTP induction in mouse hippocampal slices (Ji et al., 2001). Local puff application of ACh to the apical dendrites was sufficient to boost short term plasticity of Schaffer collateral synapses to LTP. But when the same stimulus was delayed until nAChR-mediated GABAergic inhibition reached the pyramidal neuron, LTP was prevented. In addition to nAChR activity, mAChR activation was shown to modulate the induction and amplitude of LTP at hippocampal Schaffer collateral synapses in slice preparations from rats (Huerta and Lisman, 1995; Buchanan et al., 2010) or mice (Shinoe et al., 2005). Similar results were obtained when cholinergic activity was evoked by tail pinch or electrical stimulation of the medial septum nuclei in anesthetized rats *in vivo* (Navarrete et al., 2012). Induction of LTP was blocked in this study by systemic atropine application, confirming the contribution of mAChRs. Mechanistically, Buchanan et al. (2010) showed that postsynaptic activation of the muscarinic M1 receptor subtype resulted in the inhibition of SK channels, allowing enhanced NMDA receptor activity and eventually leading to a facilitation of LTP induction.

Cholinergic receptor activation also enhances spike backpropagation, and thereby affects the amplitude and duration of spike train-evoked Ca^2+^ changes in apical dendrites, thus affecting synaptic integration and plasticity (Tsubokawa and Ross, 1997).

## Acetylcholine Effects on Astrocytes

Besides its effects on neurons, ACh also acts on astrocytes. Calcium imaging from acute rat hippocampal slices demonstrated the presence of functional α7-containing nAChRs on astrocytes in the CA1 (Shen and Yakel, 2012) and CA3 region (Grybko et al., 2010). Although the current density is very low, the calcium response upon receptor activation is robust due to the calcium induced calcium release from the endoplasmic reticulum mediated via inositol trisphosphate (IP3) receptor activation (Sharma and Vijayaraghavan, 2001; Grybko et al., 2010). In contrast to these studies, optogenetic stimulation of ACh release from CA1 cholinergic fibers in rat hippocampal slice preparations did not reveal significant nicotinic receptor-mediated effects, but instead mobilized Ca^2+^ from intracellular stores via muscarinic receptor activation (Araque et al., 2002). In this study, different regions in the recorded astrocytes showed independent stimulus-induced Ca^2+^ variations, suggesting the existence of subcellular domains in the astrocytic responses evoked by the synaptic cholinergic activity. One caveat of this study, however, is that the potassium channel blocker 4-aminopyridine (4-AP) was added to the slice in order to enhance synaptic release of ACh. Imaging of calcium activity in the barrel cortex of mice *in vivo* revealed that astrocytes exhibited elevated intracellular calcium levels during the induction of LTP (Takata et al., 2011). Moreover, the induction of LTP could not be induced in IP3 receptor type 2 knockout mice, indicating that calcium release from intracellular stores in astrocytes might be necessary for LTP induction. Likewise, ACh release evoked by tail pinch or electrical stimulation of the medial septum nuclei in anesthetized rats increased Ca^2+^ in hippocampal astrocytes and induced LTP at Schaffer collateral synapses, an effect dependent on mAChR activation (Navarrete et al., 2012). Further follow-up experiments performed *in vitro* confirmed the necessity of Ca^2+^ elevations in astrocytes for LTP induction at the Schaffer collateral synapse in the hippocampus, as previously shown for synapses in the barrel cortex.

Astrocytic activity has also been shown to contribute to γ oscillatory activity and disrupting gliotransmitter release from astrocytes impairs novel object recognition (Lee et al., 2014), two phenomena closely linked to cholinergic activity. Furthermore, genetic deletion of α7 nicotinic receptors causes mild but significant deficits in spatial learning (Levin, 2012). Activation of the α7 nicotinic receptor on astrocytes has also been shown recently to be involved in the regulation of the sleep-wake cycle by ACh (Papouin et al., 2017). Astrocytes in the hippocampus sense the wakefulness-dependent activity of septal cholinergic fibers through the α7-nAChR, whose activation drives D-serine release, which acts as a co-agonist at the NMDA receptor. Thus, astrocytes provide a link between cholinergic activity and NMDA receptor function. This is particularly interesting for the understanding of schizophrenia, a neurological disorder characterized by NMDAR hypofunction.

## Cholinergic Dysfunction in Neurological Disorders

Given the outstanding role of ACh for the modulation of cortical and subcortical brain regions, it is not surprising that cholinergic dysfunction is correlated with various neurological and psychiatric disorders including depression, schizophrenia, epilepsy and Alzheimer’s disease (AD). In this review we will focus our discussion on AD and epilepsy, but see Higley and Picciotto (2014) for a recent discussion of cholinergic dysfunction in depression and schizophrenia.

## Cholinergic Dysfunction in Alzheimer’s Disease

AD is the most common form of dementia in the elderly with progressive episodic memory deficits and global impairment of cognitive function at later disease states. The definitive diagnosis of AD is still based on post-mortem histophathological examinations of the patients’ brains. AD is characterized anatomically by cortical and white matter atrophy and histologically by the presence of large numbers of extracellular amyloid β (Aβ) plaques, as well as intracellular neuropil threads and neurofibrillary tangles consisting of twisted filaments of hyperphosphorylated tau protein, which also accumulates in the extracellular space after neuronal death (for review, see Serrano-Pozo et al., 2011). The extent of neurofibrillary tangles and neuropil threads found in different brain areas of post-mortem brains correlate with disease states: neurofibrillary changes are first observed in the EC, spreading to the hippocampus, and finally found in all isocortical areas correlating with neuronal damage (Braak and Braak, 1991). Given the central roles of ACh and the hippocampal formation for learning and memory, a cholinergic deficit, particularly within the hippocampal formation, has been suggested to contribute to the memory deficits observed in the elderly and particularly in AD. Supporting this hypothesis, the number of ChAT^+^ neurons was found to be reduced along the entire length of the basal forebrain in aged vs. young rats (Smith et al., 1993), and the proportion of rhythmically bursting neurons inside the MSDB was lower in aged vs. young rats, especially during immobile arousal states associated with atropine-sensitive theta activity (Apartis et al., 2000). Furthermore, cholinergic synaptic transmission in the hippocampus declines with age (Taylor and Griffith, 1993). In post-mortem tissue of AD patients, a substantial decrease of AChE and ChAT enzyme activity in many cortical areas, including the hippocampus, has been observed (Davies, 1979), indicating loss of cholinergic function at the beginning of AD. Radioligand binding assays found binding of [^3^H]-labeled nicotine to the DG granule cell layer, the presubiculum and the parahippocampal gyrus 30% reduced in post-mortem tissue of AD patients relative to age-matched elderly control subjects (Perry et al., 1995), suggesting a decrease of nAChR expression in these areas. Moreover, Aβ_1–42_ peptide was found to bind to nicotinic receptors of both the α7 and the non-α7 subtype, with higher affinity to the α7 subtype (Wang et al., 2000a,b). On a functional level, this binding has been demonstrated to inhibit nicotinic currents in rat hippocampal slices (Pettit et al., 2001) and research focused recently on the role of nicotinic AChRs, especially of the α7 subtype, for possible treatment options in AD (Vallés et al., 2014). At the moment AChE inhibitors are the most used drugs for treatment of mild to moderate AD, although they only show small benefits at the early stages of AD and do not prevent further progression of the disease (Kaduszkiewicz et al., 2005).

Nevertheless, the cholinergic deficit observed in AD can have substantial effects on the structure and thereby function of microcircuits with important consequences for cognitive processes and behavior. A recent study by Schmid et al. (2016) shows that structural plasticity of dendritic spines on O-LM interneurons is impaired in transgenic APP/PS-1 mice. This mouse line is commonly used as a model for AD. It carries mutations in the amyloid precursor protein and the presenilin-1 protein of the γ-secretase complex, a combination of mutations which lead to elevated β-amyloid production associated with cognitive impairments and memory deficits while ageing. The impairment of structural plasticity found by Schmid et al. (2016) in this mouse model was due to a loss of cholinergic input onto O-LM interneurons. Moreover, septal cholinergic input onto O-LM interneurons was shown to be necessary for fear conditioning induced spine gain on O-LM interneurons and application of cevemeline, an M1 AChR agonist, significantly improved memory deficits in the APP/PS1 mice. Thus, decreased cholinergic drive onto O-LM interneurons contributes to rewiring and memory deficits under AD-like conditions.

Previous models suggested that lower levels of ACh resulting in reduced presynaptic inhibition by muscarinic receptors could lead to excessive synaptic modification that could contribute to the progression of AD (Hasselmo, 1994), which is consistent with data showing hyperactivity in the hippocampal formation in presymptomatic AD (Quiroz et al., 2010). This framework supports the use of M4 muscarinic agonists to boost presynaptic inhibition and potentially reduce the hyperactivation in AD (Newman et al., 2012). Understanding the physiological function of the septo-hippocampal cholinergic system thus remains an important step in basic research. This applies not only for AD, but also for epilepsy.

## Cholinergic Dysfunction in Epilepsy

Epilepsy is not a singular disease entity, but a group of neurological disorders characterized clinically by an enduring predisposition to generate epileptic seizures (Fisher et al., 2005). An epileptic seizure is defined as a transient occurrence of signs and/or symptoms due to abnormal excessive or synchronous neuronal activity in the brain (Fisher et al., 2005). One widely used animal model of epilepsy in basic research is the pilocarpine-induced epilepsy model. *In vivo* application of the mAChR agonist pilocarpine (together with methyl-scopolamine to block the action of pilocarpine on AChRs in the periphery) readily induces epileptic seizures and may lead to status epilepticus, resulting in spontaneous recurrent seizures following a latent period of epileptogenesis (Friedman et al., 2007). However, under physiological conditions, septal cholinergic neurons appear to suppress seizure activity, as indicated by a study from Ferencz et al. (2001), in which the authors showed that cholinergic septo-hippocampal deafferentiation facilitated hippocampal kindling in rats. Interestingly, chronic epileptic rats show a neuronal loss in the medial and lateral septum, which is mainly due to the loss of GABAergic neurons (80%–97%), suggesting that the processing of information in the septo-hippocampal networks might be altered (Garrido Sanabria et al., 2006). In line with the hypothesized role of GABAergic MSDB neurons for pacing hippocampal theta rhythm, early deficits are observed in spatial memory and theta rhythmic activity in such chronic epileptic rats (Chauvière et al., 2009). Conversely, epileptic seizures are less frequent during behavioral states associated with hippocampal theta rhythmic activity, e.g., active wakefulness or REM sleep, and microinjections of the muscarinic agonist carbachol into the MSDB not only elicited theta rhythmic activity, but also stopped pentylenetetrazol induced facial-forelimb seizures in rats (Miller et al., 1994). Further highlighting the role of a theta rhythmic functional network state inhibiting seizure production, electrical stimulation of the MSDB at the theta frequency range had similar effects as the carbachol microinjection. Degeneration of septal neurons, as observed in AD, might also contribute to epileptic seizures, which have a very high prevalence of 10%–22% in AD patients (Mendez and Lim, 2003).

## Conclusion

As reviewed here, physiological data demonstrates robust neuromodulatory effects of the activation of muscarinic and nicotinic receptors within cortical circuits including the hippocampal formation. These modulatory effects appear important to the encoding of new information, based on changes in network circuit dynamics as reviewed in previous articles (Hasselmo, 2006). The heterogeneity of these effects on different subtypes of neurons will require future computational modeling to develop more detailed computational hypotheses of the function of these modulatory effects. These can be tested in experimental studies exploring the functional role of the strong neuromodulatory effects observed in physiological studies.

## Author Contributions

All authors conceived and wrote the review.

## Conflict of Interest Statement

The authors declare that the research was conducted in the absence of any commercial or financial relationships that could be construed as a potential conflict of interest.
